# Leaf-Specific Classification of Multi-Leaf Collimator Positioning Errors in Volumetric Modulated Arc Therapy Using a Convolutional Neural Network

**DOI:** 10.3390/jcm15135136

**Published:** 2026-07-01

**Authors:** Ju Yeol Shin, Chang Heon Choi, Jung-in Kim, Jong Min Park, Wonjoong Cheon, So-Yeon Park

**Affiliations:** 1 Paprica Lab. Co., Ltd., Seoul 03123, Republic of Korea; naturalbb123@gmail.com (J.Y.S.); dm140@snu.ac.kr (C.H.C.); madangin@snu.ac.kr (J.-i.K.); 2 Department of Radiation Oncology, Seoul National University Hospital, Seoul 03080, Republic of Korea; jongminpark@snu.ac.kr; 3 Department of Radiation Oncology, Seoul St. Mary’s Hospital, College of Medicine, The Catholic University of Korea, Seoul 06591, Republic of Korea; 4 Department of Radiation Oncology, Veterans Health Service Medical Center, Seoul 05368, Republic of Korea; 5 Institute of Radiation Medicine, Seoul National University Medical Research Center, Seoul 03080, Republic of Korea

**Keywords:** multi-leaf collimator, quality assurance, deep learning, convolutional neural network, fluence map, volumetric modulated arc therapy, MLC error classification

## Abstract

**Background/Objectives**: Multi-leaf collimator (MLC) positioning accuracy critically affects delivered dose fidelity in volumetric modulated arc therapy (VMAT), yet conventional gamma-based quality assurance (QA) provides only plan-level pass/fail outcomes without leaf-specific error localization. This study developed and validated a convolutional neural network (CNN) framework that classifies the magnitude and direction of individual MLC leaf positioning errors directly from fluence map data. **Methods**: Three patient cohorts were analyzed: 20 prostate cancer patients for model development under an 8:1:1 train/validation/test split and 20 additional prostate and 10 head and neck (H&N) patients reserved for external validation. For inner MLC leaves 21–40, systematic offsets from −5 mm to +5 mm in 1.0 mm increments were independently applied to the two leaf banks, yielding 121 error combinations per leaf. A CNN was trained as a 121-class classifier on two-channel inputs pairing the reference and error-induced fluence map regions and was compared against three tree-based baselines using five-fold cross-validation. **Results**: The CNN achieved 97.00% accuracy on the internal test set and 96.54 ± 0.43% accuracy across the five patient-level cross-validation folds. Across all test samples, 99.88% and 99.83% of predictions were within 1 mm of the true offset for Bank A and Bank B, respectively, well within the AAPM TG-142 1 mm MLC positioning tolerance. External validation yielded 96.19% accuracy on the additional prostate cohort and 93.72% on the H&N cohort, suggesting reproducibility within the same anatomical site and potential robustness across anatomically distinct treatment sites within a single-institution dataset. **Conclusions**: The proposed CNN framework demonstrates the feasibility of leaf-specific identification of MLC positioning errors in both magnitude and direction from simulated fluence maps. These findings support further investigation using physically measured fluence data for future clinical translation.

## 1. Introduction

With advancements in radiation therapy technology, techniques such as Volumetric Modulated Arc Therapy (VMAT) and Intensity-Modulated Radiation Therapy (IMRT) have been developed and widely adopted. VMAT, in particular, shapes highly conformal doses by varying the MLC aperture, dose rate, and gantry speed continuously during arc delivery, concentrating doses on the target while limiting exposure of nearby normal tissue [1,2]. However, the dynamic and simultaneous modulation of these parameters inherently introduces greater delivery uncertainty compared to conventional techniques.

Of the mechanical factors governing VMAT delivery—including gantry rotation, dose rate, and MLC positioning—errors in MLC leaf positions have been widely recognized as having a particularly significant impact on delivered dose accuracy, since the MLC directly collimates the radiation beam to define the shape and intensity of the radiation field. Even submillimeter deviations can lead to substantial discrepancies between planned and delivered doses, compromising treatment efficacy and increasing the risk of adverse effects on healthy tissues. Such errors are especially critical in VMAT, where MLC leaves must continuously reposition in synchronization with gantry rotation and dose rate modulation, leaving minimal tolerance for positioning deviations [3,4,5,6,7,8]. Patient-specific QA is therefore performed before treatment to confirm that the delivered dose agrees with the treatment planning system (TPS) calculation [3,9,10].

Delivery QA is most commonly assessed with the gamma index, which combines a dose-difference and a distance-to-agreement criterion to score how closely the measured dose matches the plan at each point. However, the gamma index is limited for error localization: it is relatively insensitive to small, localized discrepancies and collapses the spatial dose distribution into a single passing rate and a pass/fail decision, without indicating which MLC leaves are misaligned [8,9,11].

As complementary approaches, modulation indices and texture-based indices derived from modulating parameters and fluence maps have been developed to quantify treatment plan complexity and predict delivery accuracy [6,7,12,13]. Additionally, indices of achievement, which measure the alignment between planned and delivered fluences, have shown promise in improving QA reliability [14]. However, these approaches remain indirect, predicting delivery accuracy at the plan level rather than resolving errors at the individual-leaf level.

Recent advances in deep learning have further enhanced patient-specific QA. Nyflot et al. [15] and Wootton et al. [16] applied CNNs and radiomic analysis to gamma images to detect systematic and random MLC errors in IMRT, while Kimura et al. [17] extended this approach to VMAT using cylindrical detector measurements with a multi-task CNN capable of classifying multiple error types simultaneously. Nakamura et al. [18] further demonstrated that deep learning applied to MLC modeling parameters—specifically transmission factor and dosimetric leaf gap—could distinguish error types from dose difference maps with high sensitivity [18]. Beyond dose-based inputs, trajectory log-file data have also been leveraged; Carlson et al. [19], Osman et al. [20], and Chuang et al. [21] used machine learning to predict MLC positional deviations from such log-file parameters, yet log-file readings reflect only the machine’s recorded motor positions and may not reveal fluence-level discrepancies when motor encoders report nominal values. This limitation can be mitigated by fluence map-based approaches that reflect the radiation distribution actually delivered.

Despite these advances, existing approaches share a fundamental shortcoming in resolving leaf-specific delivery errors: dose- and gamma-based methods predominantly operate at the plan level through aggregate metrics or their pass/fail dichotomization, while log-file-based methods, though leaf-specific, capture only mechanical positions rather than the resulting delivered fluence [11]. Furthermore, fluence maps, which directly encode the cumulative radiation intensity shaped by MLC positions across all control points, remain largely unexplored as a primary input for leaf-specific error classification. To bridge this methodological gap, this study develops a CNN-based framework that directly analyzes fluence map data to classify MLC leaf positioning errors at the individual leaf level, simultaneously identifying both the magnitude and direction of deviations across a range of −5 mm to +5 mm as a 121-class classification problem. This level of leaf-specific error resolution has not been addressed in prior fluence-based QA studies. The proposed approach aims to provide more detailed error localization than conventional plan-level pass/fail assessments. Its robustness is further evaluated across anatomically distinct treatment sites, while broader validation across institutions, treatment planning systems, and MLC platforms remains necessary for future clinical translation.

## 2. Methods

### 2.1. Study Design and Patient Cohorts

This retrospective study (approved by the Institutional Review Board of the Veterans Health Service Medical Center, approval number: 2022-01-002-005) analyzed VMAT treatment planning data from three independent patient cohorts (Table 1): a primary prostate cohort (*n* = 20) for model development under an 80%/10%/10% training/validation/test split, an additional prostate cohort (*n* = 20) for external validation within the same anatomical site, and a head and neck (H&N) cohort (*n* = 10) for cross-site generalizability assessment.

Patient-level data partitioning was used throughout all experiments to prevent data leakage between training and evaluation datasets. Specifically, all samples generated from a given patient were assigned exclusively to either the training, validation, or test set. Consequently, no patient contributed samples to more than one subset.

The internal dataset consisting of 20 prostate cancer patients was divided using an 8:1:1 patient-level split, corresponding to 16 patients for training, 2 patients for validation, and 2 patients for testing. Because multiple fluence-map samples were generated from each patient, this strategy ensured that the model was evaluated on entirely unseen patients rather than unseen samples from previously observed patients.

All treatment plans comprised two full arcs and were delivered using the Varian High-Definition (HD) MLC system (Varian Medical Systems, Palo Alto, CA, USA), which consists of 60 leaf pairs—each pair comprising two opposing leaves from Bank A and Bank B—with leaf widths of 5 mm for outer leaves (leaves 1–14 and 47–60) and 2.5 mm for inner leaves (leaves 15–46) at the isocenter. A summary of the study design is provided in Table 1.

The VMAT treatment plans were retrospectively collected from clinically approved treatment plans generated for routine patient care. All plans were created using the institutional treatment planning workflow and exported as DICOM RT Plan files for subsequent fluence-map reconstruction and error simulation.

**Figure 1:**
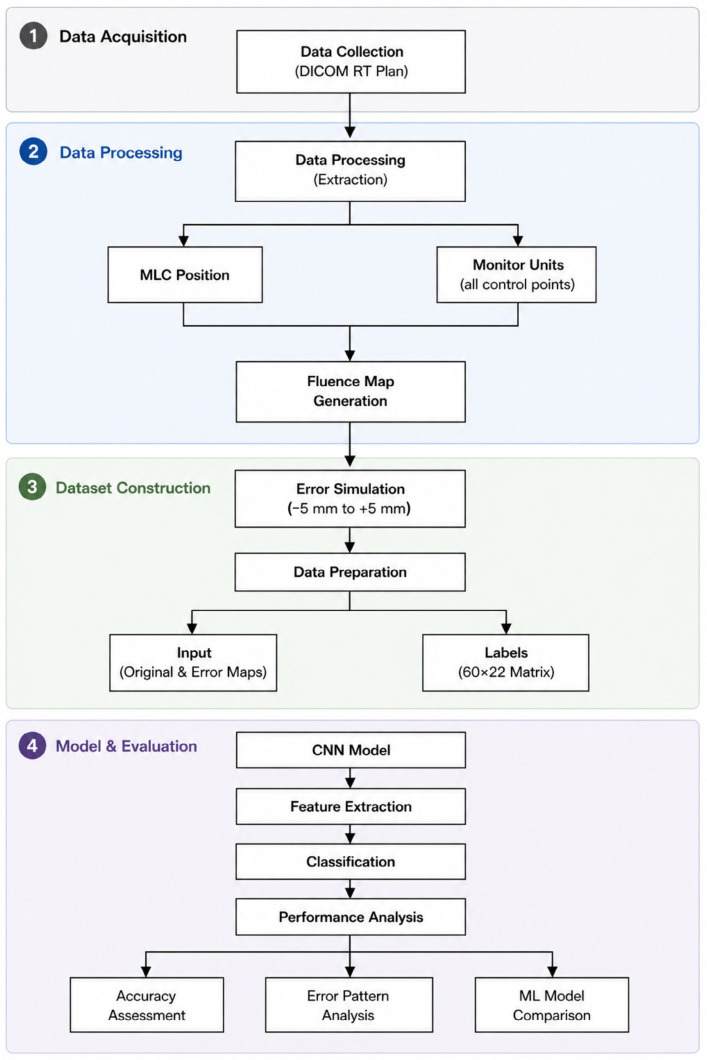
Overview of the data processing and deep learning model workflow for MLC error detection.

The overall workflow of the proposed framework—encompassing data preparation, fluence map generation, MLC error simulation, model training, and performance evaluation—is summarized in Figure 1. Each methodological component is described in detail in the following sections.

### 2.2. Fluence Map Generation and ROI Extraction

For each treatment plan, fluence maps were generated from the Digital Imaging and Communications in Medicine (DICOM) RT plan file through a three-step process: (1) extraction of MLC positions and monitor units (MUs) at all control points; (2) processing of leaf positions from both MLC banks; and (3) accumulation of MLC-shaped beam contributions weighted by the corresponding MUs to produce the composite fluence map. The resulting maps represented the cumulative radiation intensity distribution delivered over the entire arc, at a spatial resolution of approximately 1.0 mm per pixel.

From each fluence map reconstructed at the isocenter plane, a leaf-specific region of interest (ROI) was extracted to serve as input for the deep learning model.

ROI extraction was performed automatically using the geometric leaf-position information contained in the DICOM RT plan. Because the ROI coordinates were deterministically defined from the planned MLC leaf locations, no manual contouring, expert annotation, or user-dependent intervention was required. Consequently, the ROI generation process was free from inter-observer and intra-observer variability.

Analysis was restricted to inner MLC leaves 21–40. Although the HD120 MLC contains 32 high-resolution central leaves (leaves 15–46, projected width 2.5 mm at isocenter), leaves 21–40 occupy the geometric center of the treatment field and are most frequently involved in aperture formation for prostate and head-and-neck VMAT plans. Consequently, these leaves exhibit the highest modulation activity and contribute most substantially to the delivered fluence. In contrast, the more peripheral high-resolution leaves (15–20 and 41–46) more frequently remain outside the treatment aperture or behind the jaws during beam delivery. Restricting the analysis to leaves 21–40 therefore ensured that introduced positioning errors consistently generated measurable perturbations in the fluence maps, thereby maximizing both the dosimetric relevance of the simulated errors and the quality of the resulting training data.

Because each leaf was analyzed independently using an identical ROI definition and network architecture, the proposed framework is inherently leaf-agnostic. The selection of leaves 21–40 was therefore intended as a proof-of-concept scope focusing on the most clinically relevant high-resolution leaves rather than a methodological limitation. Extension to the remaining high-resolution leaves or the full MLC bank would not require modification of the proposed CNN architecture and therefore represents a natural direction for future work.

To represent each leaf consistently, an ROI of three vertical pixels by 400 horizontal pixels was defined. At the isocenter level, the inner leaves have a projected width of 2.5 mm; the three-pixel vertical extent was chosen to fully encompass this leaf width while providing a uniform input size across all leaves, and the 400-pixel lateral extent covered the full range of leaf travel within the treatment field.

**Figure 2:**
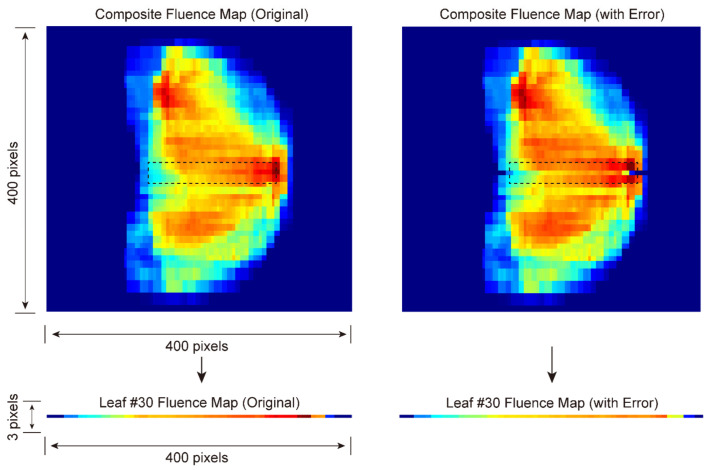
Fluence map generation and leaf-specific ROI extraction. (**Upper**) Original and error-induced composite fluence maps. The color scale represents relative fluence intensity, with blue indicating lower fluence and red/yellow indicating higher fluence. (**Lower**) Extracted ROIs for MLC leaf #30, consisting of three vertical pixels and 400 horizontal pixels. The dotted rectangles indicate the selected leaf-specific ROIs used for model input. These segments are paired and stacked to form a model input of size 3 × 400 × 2.

For model input, the ROI from the reference fluence map and the ROI from the corresponding error-induced fluence map were stacked to form a two-channel tensor of shape 3 × 400 × 2. This paired representation enabled the model to learn from the direct comparison between reference and error-induced fluence distributions rather than from a single fluence map in isolation. Figure 2 illustrates the fluence map generation and ROI extraction process, showing the reference and error-induced composite fluence maps together with the extracted ROI for MLC leaf #30.

### 2.3. MLC Error Simulation and Label Encoding

To generate supervised learning labels, synthetic MLC positioning errors were introduced directly into the MLC leaf coordinates extracted from the DICOM RT plan files. For each error configuration, the modified leaf positions were used to regenerate an error-induced fluence map while preserving the original monitor-unit weighting and control-point sequence. The resulting reference and error-induced fluence maps formed a paired sample for model training.

Error simulation was applied to the inner MLC leaves (leaves 21–40), which shape the central treatment field in central-target VMAT plans and represent the most clinically relevant region for positioning error detection. Systematic offsets ranging from −5 mm to +5 mm in 1.0 mm increments were independently applied to Bank A and Bank B, yielding 11 discrete error states per bank and 121 unique error combinations (11 × 11) per leaf. This design ensured uniform representation of all possible error states across the 121-class classification task, with each combination treated as a distinct class so that both the magnitude and direction of deviations in the two banks could be identified simultaneously.

Leaf-specific positioning errors were defined as translational offsets applied independently to the corresponding leaves of Bank A and Bank B. Positive values represented outward leaf displacement, whereas negative values represented inward displacement relative to the planned position. Because each bank could assume one of 11 discrete offset states (−5 mm to +5 mm in 1 mm increments), each leaf was characterized by a unique pair of Bank A and Bank B offsets. Consequently, the error classification problem was formulated as a 121-class task (11 × 11), where each class corresponded to a distinct combination of leaf-position deviations in the two banks. For example, a leaf with a +3 mm offset in Bank A and a −1 mm offset in Bank B constituted a different class from a leaf with a +3 mm offset in Bank A and a 0 mm offset in Bank B, allowing simultaneous identification of both error magnitude and direction.

**Figure 3:**
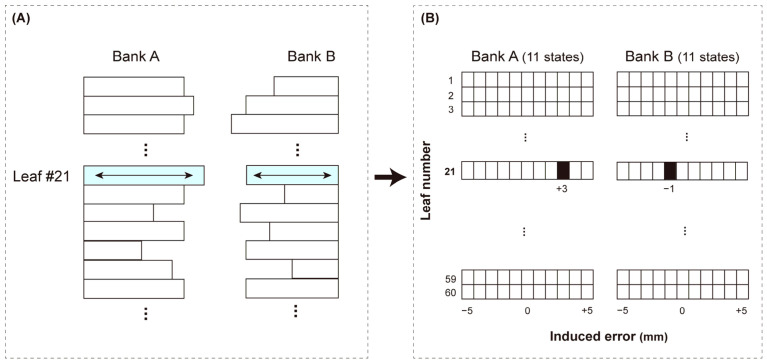
**MLC error simulation and 121-class label encoding.** (**A**) For each analyzed leaf (shown here for Leaf #21), positioning errors are applied independently to the Bank A and Bank B leaves, each spanning −5 mm to +5 mm in 1-mm steps (11 discrete states per bank). The blue shading indicates the selected leaf, and the double-headed arrows indicate the direction of the simulated leaf displacement. Ellipses indicate omitted intermediate leaves for schematic clarity. (**B**) Ground-truth label representation in which each leaf occupies one row. The marked cell in the Bank A block (**left**, 11 states) and in the Bank B block (**right**, 11 states) indicates that leaf’s Bank A and Bank B error states, respectively (example: Leaf #21 with Bank A = +3 mm and Bank B = −1 mm). The filled black cells represent the induced error states used for label encoding. Although displayed as two 11-state blocks for readability, the CNN was trained as a 121-class classifier in which each leaf’s (Bank A, Bank B) pair corresponds to one of the 11 × 11 = 121 joint error classes. Error simulation was applied to the inner leaves 21–40 (see Section 2).

For each sample, the ground truth was encoded as a categorical label corresponding to one of the 121 error combinations, which directly mapped to the 121-unit softmax output of the CNN, enabling end-to-end multi-class classification. An illustrative example is shown in Figure 3: when a +3 mm offset is applied to Bank A and a −1.0 mm offset to Bank B, the corresponding combination is encoded as the class index assigned to this pairing within the 121-class scheme. For each analyzed leaf, all 121 error combinations were generated independently, resulting in 2420 samples per leaf for the primary prostate cohort (20 patients × 121 error combinations). Across the 20 analyzed leaves, this yielded a total of 48,400 samples for model development.

Because all 121 error combinations were generated systematically for every analyzed leaf and patient, the resulting classification dataset was inherently balanced, with identical numbers of samples represented in each class. Therefore, no additional class weighting, oversampling, or undersampling procedures were required during model training.

### 2.4. Deep Learning Model Architecture and Optimization

Prior to model training, the reference and error-induced fluence maps were converted into paired leaf-specific ROIs as described above. For each sample, the ROI extracted from the reference fluence map and the corresponding ROI extracted from the error-induced fluence map were stacked along the channel dimension to form a two-channel input tensor. The resulting input size was 3 × 400 × 2, where the first channel represented the reference fluence distribution and the second channel represented the error-induced fluence distribution. No additional image augmentation was applied. The dataset was subsequently partitioned into training, validation, and test sets using patient-level splitting to prevent data leakage between cohorts. No additional fluence normalization was applied. The reference and error-induced fluence maps were directly used for ROI extraction and model training because both maps were generated using the same fluence-map generation procedure.

**Figure 4:**
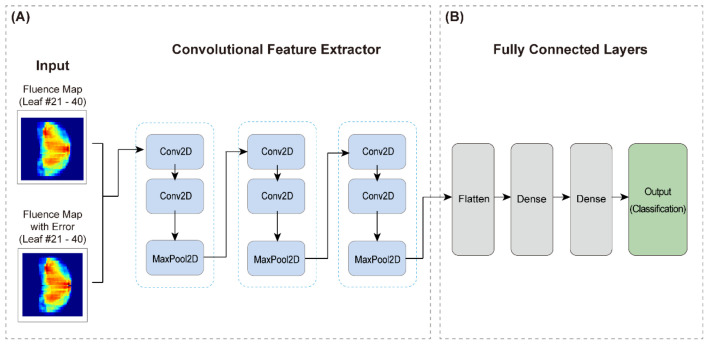
CNN architecture for MLC error detection and classification. (**A**) Feature extraction using convolutional and pooling layers. (**B**) Classification using fully connected layers. The colored network blocks distinguish layer types: light blue indicates convolutional and pooling layers, gray indicates fully connected layers, and green indicates the output layer.

A CNN architecture was developed to classify MLC positioning errors from the paired fluence map ROI defined in the preceding sections. The network received an input tensor of shape 3 × 400 × 2, where the two channels corresponded to the reference and error-induced fluence map segments, enabling the model to learn from the direct comparison between the two states rather than from a single fluence distribution. The architecture is illustrated in Figure 4. The feature extraction backbone consisted of six convolutional layers organized into three sequential blocks, with each block comprising two convolutional layers with a kernel size of 1 × 3 followed by a max-pooling layer with a pool size of 1 × 4 to progressively reduce the lateral dimension. This asymmetric kernel design was intentionally selected to perform convolution primarily along the lateral 400-pixel axis, where MLC leaf displacement produces the dominant fluence modulation pattern. In contrast, the vertical dimension of the input ROI consisted of only three pixels and was used mainly to encompass the projected leaf width at the isocenter; therefore, applying additional vertical convolution or pooling was not expected to provide meaningful spatial feature extraction and could instead reduce the already limited vertical information.

The hierarchical convolutional structure enabled the network to progressively capture fluence perturbation patterns produced by MLC positioning errors. Earlier layers learned localized differences between the reference and error-induced fluence maps, whereas deeper layers aggregated these local patterns into higher-level spatial representations associated with specific combinations of Bank A and Bank B leaf offsets. Progressive pooling further increased the effective receptive field along the lateral axis while reducing feature dimensionality.

The number of filters increased with depth and was determined through hyperparameter optimization, with block 1 containing 16 and 32 filters, block 2 containing 64 and 64 filters, and block 3 containing 128 and 128 filters. All convolutional layers used ReLU activation and “same” padding to preserve spatial dimensions, and batch normalization and dropout (rate = 0.2) were applied after each convolutional layer to stabilize training and reduce overfitting. The output of the final block was flattened and passed through a dense layer with 512 units and ReLU activation, followed by the final output layer of 121 units with softmax activation, corresponding to the 121 error combinations defined in the MLC Error Simulation and Label Encoding section.

Model hyperparameters were optimized using Keras Tuner with a RandomSearch strategy. The search space included the number of additional convolutional layers (1–3), the number of filters in these layers (64 or 128), the number of units in the fully connected layer (128, 256, 384, or 512), and the learning rate (1 × 10^−2^, 1 × 10^−3^, and 1 × 10^−4^). A total of 15 search trials were conducted, and the architecture yielding the highest validation accuracy was selected as the final model. The kernel size (1 × 3), pooling size (1 × 4), activation function, output formulation, and batch size were fixed throughout the optimization process. The final model was trained using the Adam optimizer and categorical cross-entropy loss with a batch size of 8 for a maximum of 20 epochs. Network weights were randomly initialized, and the dataset partition used a fixed random seed (random_state = 42) to ensure reproducible splits.

No data augmentation techniques were applied. Conventional image augmentations such as rotation, flipping, or geometric transformation were intentionally avoided because they would alter the physical correspondence between fluence-map patterns and the underlying MLC positioning errors.

Early stopping (patience = 10 epochs) did not trigger within the 20-epoch training budget, so the mechanism effectively reduced to best-validation-checkpoint selection. Models were trained for the full 20 epochs, and the parameter set corresponding to the lowest validation loss was retained for final evaluation; all reported performance metrics were thus derived from the best-validation checkpoint rather than from the final training epoch.

All experiments were implemented in TensorFlow 2.10.0 and executed on an NVIDIA GeForce RTX 4090 GPU with CUDA Toolkit 11.8.

### 2.5. Implementation of Traditional Machine Learning Models for Comparison

Three tree-based machine learning models—Random Forest, XGBoost, and CatBoost—were implemented as baselines for comparison with the CNN. All three are widely used classifiers for tabular data and were selected to provide a representative benchmark from traditional machine learning methods. To ensure a fair comparison, the same two-channel fluence map ROI used as CNN input was flattened into a 2400-dimensional feature vector (3 × 400 × 2) and provided to each baseline model.

The Random Forest classifier was configured with 500 trees and a maximum depth of 10, with bootstrap aggregation and feature randomization applied to reduce overfitting. XGBoost was trained with 500 boosting iterations and a learning rate of 0.1, and CatBoost was implemented with parameters matched to those of XGBoost for consistency, leveraging its ordered boosting approach to reduce target leakage. Hyperparameters for all three models were selected through grid search. For the two gradient boosting models, L1 and L2 regularization on leaf weights were applied to stabilize training, and early stopping based on validation performance was employed for XGBoost and CatBoost to prevent overfitting.

### 2.6. Performance Evaluation

Model performance was evaluated using four standard classification metrics: accuracy, precision, recall, and F1-score. Given the 121-class nature of the task, precision, recall, and F1-score were computed using a one-vs-rest strategy and then macro-averaged across all 121 classes, ensuring equal weighting of each class regardless of its frequency in the dataset. In this formulation, true positives (TP) denote correctly identified error combinations, true negatives (TN) denote correctly rejected non-target classes, false positives (FP) denote incorrectly predicted error combinations, and false negatives (FN) denote missed error combinations.

The four metrics were defined as follows:

Accuracy=(TP+TN)/(TP+TN+FP+FN)

$$Precision=TP/\left(TP+FP\right)$$

Recall=TP/(TP+FN)

F1-score=2×(Precision×Recall)/(Precision+Recall)

In the context of medical error detection, aggregate classification accuracy alone may be insufficient because not all misclassifications have the same clinical implication. For example, a prediction that differs from the true offset by 1 mm is clinically different from a prediction that differs by several millimeters. Therefore, in addition to accuracy, precision, recall, and F1-score, deviation-based metrics were used to quantify the magnitude of prediction errors in each MLC bank. Each prediction was produced from a single leaf’s one-dimensional fluence profile (one leaf per forward pass); gradient-based attribution maps (input-gradient saliency and Grad-CAM++) are therefore interpreted as intra-leaf decision regions, whereas error localization across the MLC was obtained by applying the model to every leaf. All illustrative cases were drawn from the fixed hold-out test set (train_test_split, test_size = 0.1, random_state = 42; test *n* = 4840).

To further characterize classification reliability beyond aggregate metrics, a prediction mismatch analysis was performed. For each misclassified sample, the absolute deviation between the predicted and true offset values was computed independently for Bank A and Bank B across the full range of −5 mm to +5 mm. This analysis quantified not only whether misclassifications occurred, but also how far the predicted error magnitudes deviated from the ground truth, providing a clinically interpretable measure of model reliability.

### 2.7. Statistical Methods

To compare CNN performance against each of the three baseline machine learning models (Random Forest, XGBoost, and CatBoost), paired *t*-tests were performed on the fold-wise accuracies obtained from the five-fold cross-validation procedure. To control the family-wise error rate across the three pairwise comparisons, the Holm–Bonferroni correction was applied, with statistical significance defined as a corrected *p*-value below 0.05. Effect sizes were quantified using Cohen’s *d* to assess the practical magnitude of the observed performance differences alongside statistical significance. All analyses were conducted in Python 3.8.18 using SciPy 1.10.1 and scikit-learn 1.3.0. Because the five cross-validation folds were derived from overlapping training data, the resulting *p*-values were interpreted descriptively rather than as strictly independent inferential tests, and the effect sizes were used as the primary indicator of the magnitude of the performance differences.

### 2.8. Multi-Leaf Perturbation Scenarios for Robustness Evaluation

To investigate the impact of neighboring leaf perturbations, an additional multi-leaf error dataset was generated. In this dataset, positioning errors were simultaneously applied to the target leaf and its two immediate neighboring leaves (target leaf ±1). Two perturbation scenarios were considered. In Scenario 1, neighboring leaves were shifted in the same direction as the target leaf. In Scenario 2, neighboring leaves were shifted in the opposite direction. For these experiments, the fluence-map region of interest (ROI) was expanded from the original target-leaf-only representation to a 9-pixel vertical window encompassing the target leaf and its adjacent leaves. The ground-truth label remained defined by the target leaf error state, while neighboring leaf offsets were treated as additional perturbations for robustness evaluation.

### 2.9. Implementation of Classification and Regression Frameworks

To investigate whether MLC error identification is better formulated as a classification or regression problem and to provide a fair comparison with non-spatial neural networks, three additional models were implemented in addition to the original CNN classifier.

The original CNN classifier was retained as the primary model. For the CNN regression model, the convolutional backbone architecture was kept identical to that of the CNN classifier, including all convolutional layers, pooling layers, and the fully connected layer. Only the prediction head and loss function were modified. Specifically, the final softmax classification layer was replaced with a two-node linear output layer representing continuous positional offsets for Bank A and Bank B, and the model was optimized using mean absolute error (MAE) loss instead of categorical cross-entropy loss. This design ensured that the comparison between classification and regression formulations was performed using the same feature extraction architecture. The CNN regression model used the same architecture and hyperparameters selected for the CNN classifier; only the output layer and loss function were changed, and the model was trained from random initialization without a separate hyperparameter search.

To evaluate the importance of spatial feature learning, a multilayer perceptron (MLP) baseline was also implemented. The MLP received the same input data as the CNN models; however, the 3 × 400 × 2 fluence map ROI was flattened into a one-dimensional vector of 2400 features. Therefore, the MLP had access to the same information content but could not explicitly exploit local spatial relationships through convolutional operations.

For the MLP classifier, the flattened input was passed through fully connected layers followed by a softmax output layer corresponding to the 121 MLC error classes. For the MLP regression model, the same architecture was used except that the final softmax layer was replaced by a two-node linear output layer predicting continuous Bank A and Bank B offsets. Hyperparameter optimization was performed using Keras Tuner Random Search with 15 trials. The search space included the number of hidden layers (1–3), the number of hidden units (128, 256, or 512), the dropout rate (0.0, 0.3, or 0.5), and the learning rate (1 × 10^−2^, 1 × 10^−3^, and 1 × 10^−4^). Early stopping with a patience of 10 epochs was applied to all models.

For all experiments, identical training, validation, and test splits were used to ensure fair comparison between model architectures and learning objectives.

## 3. Results

### 3.1. Model Training Performance

On the internal test set of the primary prostate cohort, the CNN model achieved a test accuracy of 97.00%, with a precision of 97.14%, a recall of 97.06%, and an F1-score of 97.04% (Table 2). The balanced performance across these four metrics indicates that the model did not exhibit systematic bias toward any particular error class.

**Figure 5:**
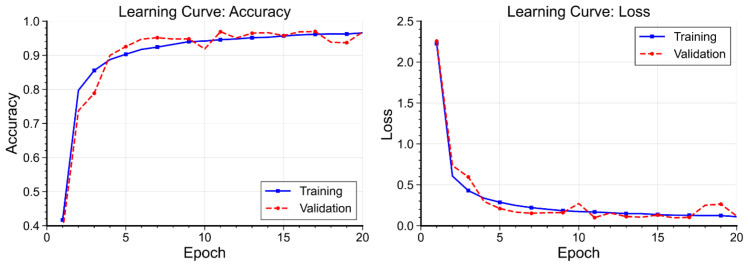
Model learning curves over 20 epochs. (**Left**) Accuracy and (**Right**) loss curves for training and validation sets. The close alignment between training and validation curves indicates robust model generalization without overfitting.

The training process showed stable convergence. After 20 epochs, the model reached a training accuracy of 97.10% and a validation accuracy of 96.81%, with close alignment between training and validation curves for both accuracy and loss (Figure 5), suggesting that the model generalized well without overfitting.

### 3.2. Cross-Validation Assessment

**Figure 6:**
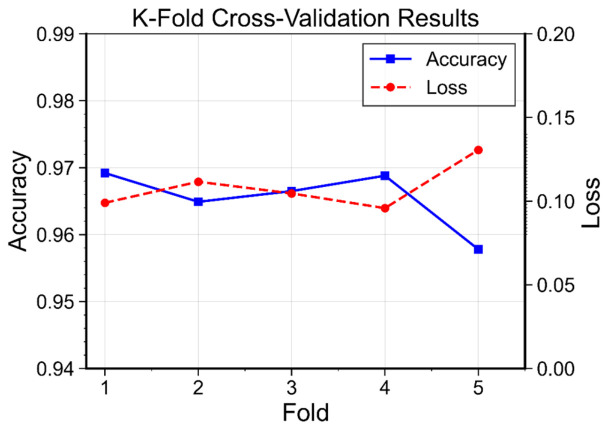
Five-fold cross-validation results for accuracy and loss.

Five-fold cross-validation confirmed model stability across different data partitions. The average cross-validation accuracy was 96.54 ± 0.43%, and the average loss was 0.1083 (Figure 6). The narrow standard deviation of 0.43 percentage points across all five folds indicates that the reported performance was not sensitive to the specific choice of training–validation split, supporting the reliability of the internal test results.

### 3.3. Comparative Analysis with Traditional Machine Learning

The CNN was compared against three tree-based baseline models—Random Forest, XGBoost, and CatBoost—trained and evaluated on identical data splits. The results revealed a substantial generalization gap in the baseline models (Table 3). XGBoost and CatBoost achieved near-perfect training accuracies of 99.92% and 99.60%, respectively, but their test accuracies dropped to 69.86% and 63.95%. Random Forest showed the most pronounced generalization failure, with accuracy decreasing from 94.29% in training to 22.00% on the test set. In contrast, the CNN maintained nearly identical accuracies on training (97.10%) and test (97.00%) sets, with a gap of only 0.10 percentage points.

Statistical comparison further confirmed the superiority of the CNN. Paired *t*-tests on fold-wise accuracies from the five-fold cross-validation yielded consistent differences between the CNN and each baseline (CNN vs XGBoost: *p* = 2.3 × 10^−7^; CNN vs Random Forest: *p* = 9.6 × 10^−9^; CNN vs CatBoost: *p* = 1.4 × 10^−7^), with all corrected *p*-values remaining below 0.001 after Holm–Bonferroni correction (Table 4). Cohen’s *d* values of 44.8, 99.6, and 43.0 for the three comparisons were classified as extremely large effects. Because the five cross-validation folds shared overlapping training data, these *p*-values are reported descriptively rather than as strictly inferential tests; the consistently large effect sizes indicate that the advantage of the CNN over every baseline was substantial across all folds.

### 3.4. Model Reliability Assessment

To characterize the precision of individual predictions beyond aggregate classification metrics, a deviation analysis was performed on the internal test set. Of the 4840 test samples, 145 (3.00%) were misclassified. For these misclassified samples, the mean deviation magnitudes remained small—0.52 mm for Bank A and 0.72 mm for Bank B—although the maximum deviation reached 5 mm in both banks (Table 5). When evaluated across all 4840 test samples, 99.88% of predictions for Bank A and 99.83% for Bank B were within 1 mm of the true offset, indicating that most prediction errors remained clinically small even when exact class matching was not achieved.

The distribution of deviation magnitudes among the 145 misclassified samples further illustrates the model’s precision (Table 6). For Bank A, 95.8% of misclassified predictions fell within a 1.0 mm deviation of the true offset (55.1% exact matches and 40.7% within 1.0 mm), while 94.5% of misclassified predictions for Bank B fell within the same threshold (35.9% exact matches and 58.6% within 1.0 mm). These results are consistent with the 1.0 mm MLC positioning tolerance recommended by AAPM TG-142 [22].

### 3.5. External Validation

To assess the generalizability of the CNN beyond the primary prostate cohort, the trained model was evaluated on two independent external datasets: an additional prostate cohort of 20 patients and a head and neck (H&N) cohort of 10 patients. Both datasets were generated using the same error simulation and sampling procedure described in Methods, and neither was used in any stage of model development.

On the additional prostate cohort, which yielded 48,400 samples (2420 per leaf × 20 inner leaves), the model achieved an accuracy of 96.19%, with a precision of 96.17%, a recall of 96.79%, and an F1-score of 96.11% (Table 7). These results were nearly identical to those obtained on the internal test set (97.00% accuracy), with a difference of approximately 1 percentage point, indicating that model performance was reproducible across independent patient cohorts within the same treatment site.

On the H&N cohort, which yielded 24,200 samples (1210 per leaf × 20 inner leaves), the model achieved an accuracy of 93.72%, with a precision of 94.34%, a recall of 93.71%, and an F1-score of 93.78% (Table 8). Although the accuracy decreased by approximately 3.5 percentage points compared to the internal prostate test set—an expected reduction given the anatomical and geometric differences between the two treatment sites—the model retained high classification performance, supporting its applicability across anatomically distinct sites. Per-patient accuracy on the H&N cohort ranged from 88.10% to 97.52% (mean 93.70% ± 3.02%; Table 9), confirming that the modest accuracy reduction was consistent across individual patients rather than being driven by a few outliers.

### 3.6. Robustness Evaluation Under Multi-Leaf Perturbation Conditions

To evaluate robustness under clinically more realistic multi-leaf perturbation conditions, additional experiments were performed using an expanded 9-pixel ROI and simultaneous errors in the target leaf and its immediate neighboring leaves. In Scenario 1, neighboring leaves shifted in the same direction as the target leaf, whereas in Scenario 2 they shifted in the opposite direction.

The CNN achieved a test accuracy of 95.20% under Scenario 1 and 96.07% under Scenario 2, compared with 97.00% for the original single-leaf dataset. Despite the increased complexity introduced by neighboring leaf perturbations, the model maintained high classification performance, suggesting that the proposed framework remains robust in the presence of correlated multi-leaf positioning errors (Table 10).

### 3.7. Comparison of Classification and Regression Frameworks

To compare classification and regression formulations, additional CNN and MLP regression models were trained and evaluated using the same dataset and train–validation–test splits.

The CNN regression model achieved a test MAE of 0.071 mm (Table 11). The per-bank MAE (Mean Absolute Error) values were 0.062 mm for Bank A and 0.080 mm for Bank B. After rounding continuous predictions to the nearest integer-millimeter error state, the exact-match accuracy was 96.82%. Furthermore, 99.96% and 99.81% of predictions were within 1 mm for Bank A and Bank B, respectively. Most predictions exhibited zero deviation from the ground truth after rounding, indicating highly accurate estimation of continuous MLC offsets.

For the non-spatial baseline, the MLP classifier achieved a test accuracy of 97.11%, which was comparable to that of the CNN classifier. Hyperparameter optimization selected two hidden layers with 512 neurons each, a dropout rate of 0.3, and a learning rate of 1 × 10^−4^. The resulting model achieved a macro-averaged precision, recall, and F1-score of approximately 97%.

In contrast, the MLP regression model showed substantially lower performance than the CNN regression model. The optimized MLP regression architecture consisted of two hidden layers with 512 neurons each, a dropout rate of 0.3, and a learning rate of 1 × 10^−4^. The model achieved a test MAE of 0.374 mm, with per-bank MAE values of 0.383 mm and 0.365 mm for Bank A and Bank B, respectively. The exact-match accuracy after rounding was 61.57%, although more than 98% of predictions remained within 1 mm of the true error magnitude.

Overall, the results demonstrate that both classification and regression formulations can successfully identify MLC positioning errors. However, the substantial performance gap between CNN regression and MLP regression suggests that spatial feature extraction plays a critical role when estimating continuous leaf displacements.

## 4. Discussion

**Figure 7:**
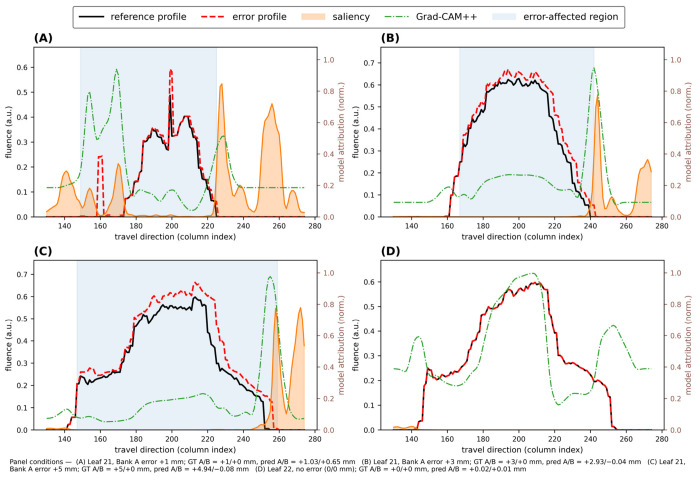
Intra-leaf decision region of the per-leaf CNN. Each panel shows a single leaf—the model’s actual input—for four hold-out test cases: (**A**) Bank A +1 mm, (**B**) +3 mm, (**C**) +5 mm single-leaf errors, and (**D**) an error-free case. Black solid and red dashed curves are the reference and error one-dimensional fluence profiles; the shaded band marks the error-affected columns. Overlaid (right axis) are the model’s input-gradient saliency (orange) and Grad-CAM++ (green, computed at conv2d_3) for the predicted offset of the affected bank. Both attributions concentrate on the displaced leaf edge, showing that the network bases its decision on the shifted leaf boundary; in the error-free case the attribution is low and diffuse.

This study developed a CNN-based framework for leaf-specific classification of MLC positioning errors directly from fluence map data. On the internal test set of the primary prostate cohort, the model achieved an accuracy of 97.00% across the 121-class problem. When evaluated across all test samples, 99.88% and 99.83% of predictions were within 1 mm of the true offset for Bank A and Bank B, respectively, indicating that most residual errors remained clinically negligible. Failure-case analysis further showed that most misclassifications were clinically adjacent to the correct error state rather than large deviations. Among the 145 misclassified samples, 95.8% of Bank A predictions and 94.5% of Bank B predictions were within 1 mm of the true offset, suggesting that most errors reflected boundary ambiguity between neighboring 1-mm classes rather than complete failure of leaf-specific localization. The intra-leaf decision regions of the network, visualized with input-gradient saliency and Grad-CAM++, confirmed that predictions were driven primarily by the displaced leaf edge (Figure 7).

According to AAPM TG-142 recommendations, MLC positioning tolerances are typically on the order of 1–2 mm depending on the treatment technique and QA procedure. Therefore, a 5 mm deviation represents a clinically significant delivery error that could potentially result in substantial dose discrepancies, particularly in highly conformal treatments such as VMAT, stereotactic radiosurgery, and hypofractionated radiotherapy. Importantly, such large deviations were observed only in a very small fraction of the misclassified samples. The majority of prediction errors remained within 1 mm of the true offset, indicating that most misclassifications were unlikely to alter the clinical interpretation of the underlying MLC error state.

Performance was maintained on an independent prostate cohort (96.19% accuracy) and decreased modestly on the H&N cohort (93.72% accuracy), indicating reproducibility within prostate VMAT plans and suggesting potential robustness across anatomically distinct treatment sites. However, all cohorts were derived from a single institution using the same Varian HD MLC system. Therefore, these findings should not be interpreted as evidence of institutional, TPS-level, beam-model, or vendor-level generalizability, and further validation across multiple institutions and treatment platforms remains necessary.

**Figure 8:**
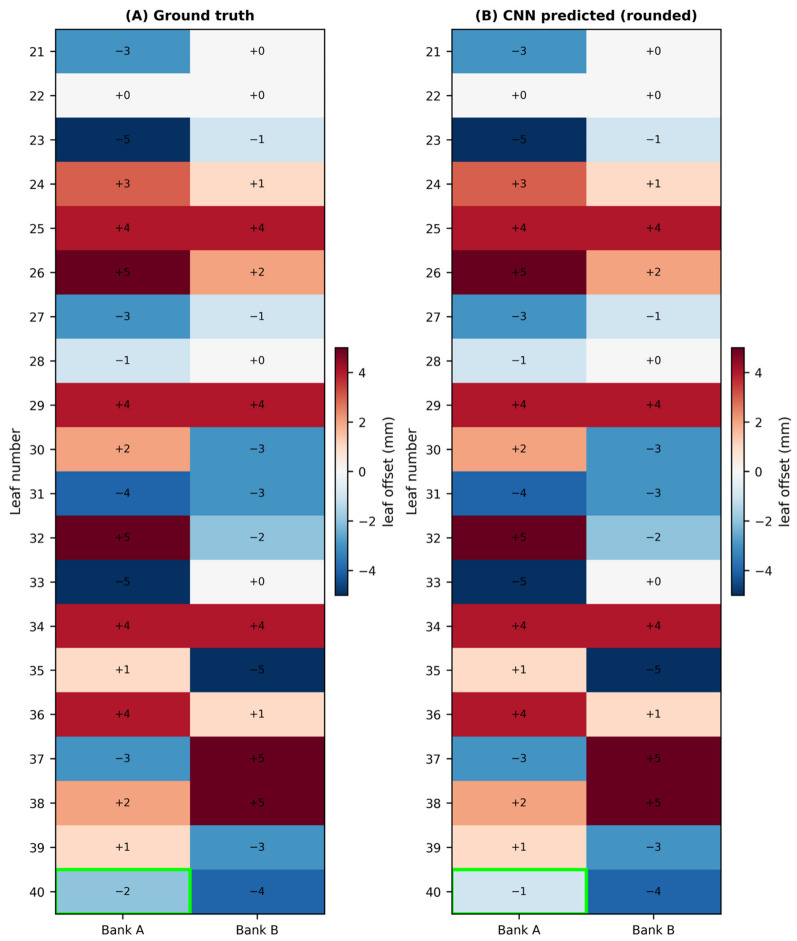
Leaf-wise error-localization map (leaves 21–40, one hold-out test case per leaf). Rows are leaf numbers; columns are Bank A and Bank B; cell color encodes the leaf offset in mm (diverging scale, −5 to +5) with the value printed in each cell. (**A**) Ground truth and (**B**) CNN prediction (rounded to integer mm). Leaves whose rounded prediction differs from ground truth in either bank are outlined in green. Because the network operates per leaf, this map identifies which leaf carries which error across the MLC—the complementary, MLC-level counterpart to the intra-leaf decision region of Figure 7. For each leaf (21–40), one of that leaf’s hold-out test cases was selected uniformly at random (seed 42), and its actual ground-truth Bank A and Bank B offsets are displayed, read jointly from a single real test case rather than drawn independently from the discrete offset states.

Beyond classification performance alone, the principal clinical contribution of the proposed framework lies in the type of information provided to the user. Conventional patient-specific QA methods, including gamma analysis, primarily assess agreement between measured and planned dose distributions at the plan level and are typically interpreted as pass/fail indicators. While such metrics are valuable for detecting the presence of delivery discrepancies, they do not directly identify which individual MLC leaves are responsible for the deviation or estimate the magnitude and direction of the underlying positioning error. In contrast, the proposed framework provides leaf-specific error localization together with quantitative error-state identification. Such information may facilitate more efficient root-cause investigation, targeted machine QA, and corrective action when delivery abnormalities are detected. Applying the trained model to every leaf produced an MLC-level error-localization map that correctly identified which leaf carried which error across the two banks (Figure 8).

Additional experiments comparing classification and regression formulations provided further insight into the nature of MLC error estimation. Because MLC positioning errors represent ordered physical quantities, regression offers a theoretically attractive framework that preserves distance information between error states. Nevertheless, the CNN regression model did not provide a meaningful performance advantage over the CNN classifier. The CNN classifier achieved 97.00% accuracy, while the CNN regression model achieved a low MAE of 0.071 mm and a rounded exact-match accuracy of 96.82%. These findings suggest that the discrete classification framework is sufficient for the current dataset, which contains predefined error states at 1-mm intervals.

The inclusion of MLP baselines clarified when spatial feature extraction matters. For the discrete 121-class task at a 1-mm resolution, the MLP and CNN classifiers were essentially indistinguishable (97.11% and 97.00% accuracy, respectively), suggesting that the 1-mm bin structure already concentrates the relevant signal into per-pixel intensity patterns that a non-spatial MLP can recover. Spatial structure became important only when continuous offsets were estimated: on the regression task the CNN substantially outperformed the MLP (MAE 0.071 versus 0.374 mm). Thus the convolutional backbone does not confer a universal advantage; it is most beneficial for continuous offset estimation, where preserving spatial relationships within the fluence map is essential, whereas for the discrete classification formulation adopted here a simpler non-spatial model performs comparably.

The large discrepancy between training and test performance observed for the XGBoost and CatBoost baselines indicates substantial overfitting. We believe this behavior is primarily attributable to the flattened tabular representation required by tree-based models. Unlike CNNs, which explicitly exploit local spatial relationships within the fluence maps, tree-based models operate on independent pixel features and therefore cannot effectively capture the spatial structure associated with MLC-induced fluence perturbations. As a result, these models tend to memorize training-specific feature combinations rather than learn generalizable spatial patterns. Importantly, the MLP baseline—although trained on the same flattened input—did not exhibit this overfitting and matched the CNN classifier, indicating that the poor generalization of the tree-based models reflects the tree-ensemble learning paradigm rather than the absence of spatial feature extraction alone. Consistent with the preceding analysis, the convolutional backbone conferred a clear advantage over the baselines for continuous offset estimation, whereas for the discrete classification task a non-spatial neural network performed comparably.

In addition, all fluence maps used in this study were analytically generated from treatment plan parameters under controlled simulation conditions. Consequently, the present work should be interpreted as a proof-of-concept study demonstrating the feasibility of leaf-specific MLC error classification rather than a clinically validated QA system. Validation using physically measured fluence data, such as EPID-acquired fluence maps, represents an important next step toward clinical translation.

Previous studies have explored deep learning for MLC error detection, yet most have operated at the plan level rather than identifying individual leaf deviations. Nyflot et al. [15], and Wootton et al. [16] applied CNNs and radiomic analysis to gamma images to detect systematic and random MLC errors in IMRT, but their frameworks relied on gamma-based inputs and produced aggregate classifications rather than leaf-level localization. Kimura et al. [17] extended deep learning to VMAT using cylindrical detector measurements with a multi-task CNN, yet their approach also aggregated predictions at the plan level. Nakamura et al. focused on MLC modeling parameters—leaf transmission and dosimetric leaf gap—derived from dose difference maps, demonstrating high sensitivity for classifying modeling-related errors but not individual positioning deviations [18]. In contrast, the present study directly analyzes fluence map data and classifies both the magnitude and direction of positioning errors at the individual leaf level, addressing a gap that these prior works have not specifically targeted.

Compared with recent deep learning-based QA studies that primarily use gamma maps, dose difference maps, detector measurements, or MLC log-file parameters as input, the present framework directly uses paired fluence-map ROIs to perform leaf-specific classification. This distinction is important because the proposed model does not merely determine whether a delivery error exists, but identifies the direction and magnitude of the error for an individual leaf.

The methodological novelty of this work has three main aspects. First, the problem was reformulated as a 121-class classification task, enabling simultaneous identification of both the magnitude and direction of leaf deviations—a formulation that, to our knowledge, has not been addressed in prior fluence-based QA studies. Second, the two-channel input representation, pairing the reference and error-induced fluence maps, allowed the model to learn from the direct comparison between the two states rather than from a single fluence distribution in isolation. This paired representation likely contributes to the model’s ability to detect subtle delivery discrepancies that machine log data—which reflect only the recorded motor positions—may not capture, even when mechanical readings appear nominal. Third, the CNN consistently outperformed the three tree-based baselines (Random Forest, XGBoost, CatBoost), with corrected *p*-values below 0.001 and Cohen’s *d* values of 43.0–99.6 for the three comparisons. The numerical magnitude of these effect sizes should be interpreted in the context of the very low fold-wise variance observed in the five-fold cross-validation (SD = 0.43 percentage points). Such tight variance, combined with the substantial accuracy gap, produced effect sizes that arithmetically exceed conventional reference ranges. While this does not imply that the accuracy difference is unprecedentedly large in a clinical sense, it does underscore that the performance advantage of the CNN was highly consistent across folds rather than driven by a few favorable splits.Several limitations should be acknowledged. First, all fluence maps used in this study were analytically generated from DICOM RT plan data rather than acquired from physical measurements. Consequently, the model was trained and evaluated under controlled simulation conditions without detector noise, scatter effects, machine-state variability, EPID acquisition artifacts, or other sources of measurement uncertainty encountered in clinical practice. Therefore, the reported performance should be interpreted as proof-of-concept evidence demonstrating the feasibility of leaf-specific error classification from fluence map information rather than as direct evidence of clinical deployment readiness. Validation using physically measured fluence data, such as EPID-acquired fluence maps with controlled MLC perturbations, represents the most important next step toward clinical translation.

Second, analysis was restricted to the inner MLC leaves (leaves 21–40), which are the most active region in central-target VMAT plans; extending the framework to outer leaves is methodologically feasible but was not evaluated in this study. Third, the primary training dataset consisted of positioning deviations applied to a single target leaf. To investigate the influence of neighboring-leaf interactions, additional robustness experiments were performed using simultaneous perturbations of the target leaf and its two immediate neighboring leaves. Two scenarios were considered: neighboring leaves shifting in the same direction as the target leaf and neighboring leaves shifting in the opposite direction. The ROI was correspondingly expanded from 3 pixels to 9 pixels to encompass all three leaves. Despite the increased complexity of the fluence patterns, the model maintained high classification performance, indicating that the proposed framework is robust to localized multi-leaf perturbations. Because the leaf-specific ROI encompasses the target leaf and only marginally overlaps its two immediate neighbors, neighboring-leaf deviations represent the perturbations most likely to directly alter the target-leaf input, whereas more distant leaves fall outside the ROI and are therefore unlikely to affect leaf-wise classification. Nevertheless, these experiments were limited to the immediate neighboring leaves and predefined perturbation patterns. Future studies should investigate more complex combinations involving larger groups of leaves and non-uniform error magnitudes to better represent realistic delivery failures. In particular, realistic MLC failures (e.g., motor wear or bank-wide calibration drift) typically involve a contiguous block of leaves with correlated but non-uniform magnitudes, which neither predefined scenario reproduces; the present evidence therefore supports robustness to localized ±1-leaf neighbor perturbations rather than to broader correlated failures. Fourth, the study focused solely on MLC positioning errors without considering other delivery uncertainties such as gantry angle, dose rate, and other mechanical parameters, which may interact with MLC errors in practice. In addition, although a formal, component-by-component ablation study was not performed, the comparison between the CNN and the multilayer perceptron (MLP) baselines—which isolates the contribution of spatial convolutional feature extraction using identical flattened inputs—together with the comparison between the classification and regression formulations provides partial ablation evidence for the two principal design choices of the proposed framework. Nevertheless, the individual contributions of the remaining components, such as the paired two-channel input, asymmetric lateral convolution, pooling strategy, and depth of the convolutional backbone, were not separately quantified. Future work should include systematic ablation experiments to determine the relative importance of these components. Finally, the 1.0 mm granularity of the simulated error states, while aligned with the TG-142 tolerance, does not resolve sub-millimeter deviations that may still carry dosimetric significance in high-precision treatments.

From a clinical workflow perspective, the proposed framework is intended as a complementary tool for patient-specific VMAT QA rather than a replacement for existing QA procedures. Following treatment plan generation, a reference fluence map and a delivery fluence map (e.g., derived from EPID measurements or reconstructed delivery data) could be processed through the same ROI extraction pipeline used in this study. The trained CNN could then perform automated leaf-by-leaf analysis and identify the most probable MLC positioning error state for each leaf. Such information could supplement conventional gamma-based QA by providing actionable localization of potential delivery errors rather than a single plan-level pass/fail metric. This leaf-specific feedback may facilitate more efficient investigation of delivery anomalies and support targeted machine QA when abnormal leaf behavior is detected.

In the present benchmark, inference required 0.552 ms per leaf ROI on a CPU-only Windows environment, corresponding to approximately 11.0 ms for analysis of the 20 leaves evaluated in this study. This suggests that CNN inference itself is not expected to be the rate-limiting step for near-real-time QA implementation, although the total workflow time would depend on fluence acquisition, reconstruction, and ROI extraction. As an immediate next step, the model should be validated against physically measured fluence data, such as EPID-acquired maps under controlled MLC perturbations, to confirm that the classification performance observed with simulated errors translates to actual delivery conditions. Evaluating the framework on multi-institutional datasets with different MLC systems would further clarify whether the learned features are specific to the Varian HD configuration or transferable across platforms. On the modeling side, training on simultaneous multi-leaf error patterns and incorporating additional delivery parameters (e.g., gantry angle and dose rate variations) are needed to better reflect the complexity of real-world delivery deviations. In the longer term, coupling this approach with real-time EPID-based in vivo dosimetry could enable online error detection during treatment delivery.

Future studies may also investigate alternative architectures, including transformer-based models and hybrid physics-informed learning approaches, to further improve robustness and generalizability.

## 5. Conclusions

This study demonstrates that a CNN trained on paired fluence map representations can classify MLC positioning errors at the individual leaf level, resolving both the magnitude and direction of deviations at a 1.0 mm resolution across a range of −5 mm to +5 mm. By providing leaf-specific, actionable information rather than plan-level pass/fail judgments, the proposed framework offers a complementary direction for patient-specific QA and a foundation for future validation with measured delivery data.

## Figures and Tables

**Table 1:** Overview of the study design and dataset composition.

Cohort	Patients	Purpose	Data Split	Samples per Leaf	Total Samples
Prostate (primary)	20	Model development	Training 80%/Validation 10%/Test 10%	2420	48,400
Prostate (additional)	20	External validation (same site)	All external test	2420	48,400
Head & Neck	10	External validation (cross-site)	All external test	1210	24,200

Scheme 121. error combinations × number of patients. Total samples = samples per leaf × 20 inner leaves (leaves 21–40). Details of error simulation and leaf selection are provided in subsequent sections.

**Table 2:** Model performance metrics on the internal test set (primary prostate cohort).

Metric	Value (%)
Accuracy	97.00
Precision	97.14
Recall	97.06
F1-score	97.04

**Table 3:** Training and test accuracies of the CNN and baseline machine learning models.

Model Type	Training Accuracy (%)	Test Accuracy (%)
CNN	97.10	97.00
XGBoost	99.92	69.86
Random Forest	94.29	22.00
CatBoost	99.60	63.95

**Table 4:** Statistical comparison of the CNN and baseline machine learning models. Because the five cross-validation folds share overlapping training data, the reported *p*-values are descriptive rather than strictly inferential.

Comparison	*p*-Value (Paired *t*-Test)	Corrected *p*-Value (Holm-Bonferroni)	Effect Size (Cohen’s *d*)	Interpretation
CNN vs. XGBoost	2.3 × 10^−7^	<0.001	44.8	Extremely large effect
CNN vs. Random Forest	9.6 × 10^−9^	<0.001	99.6	Extremely large effect
CNN vs. CatBoost	1.4 × 10^−7^	<0.001	43.0	Extremely large effect

**Table 5:** Overall model reliability on the internal test set. Mean and maximum deviation magnitudes are computed over the 145 misclassified samples.

Metric	Value
Total samples	4840
Misclassified samples	145
Misclassification rate	3.00%
Mean Bank A deviation magnitude	0.52 mm
Mean Bank B deviation magnitude	0.72 mm
Maximum Bank A deviation magnitude	5 mm
Maximum Bank B deviation magnitude	5 mm

**Table 6:** Distribution of deviation magnitudes among the 145 misclassified samples by MLC bank.

Deviation Magnitude	Bank A (%)	Bank B (%)
0 mm	55.1	35.9
1.0 mm	40.7	58.6
2 mm	2.1	4.1
3 mm	1.4	0.7
4 mm	0.0	0.0
5 mm	0.7	0.7

**Table 7:** Model performance on the additional prostate cohort.

Metric	Value (%)
Accuracy	96.19
Precision	96.17
Recall	96.79
F1-score	96.11

**Table 8:** Model performance on the head and neck cohort.

Metric	Value (%)
Accuracy	93.72
Precision	94.34
Recall	93.71
F1-score	93.78

**Table 9:** Per-patient classification accuracy on the head and neck cohort.

Patient	Accuracy (%)
1	88.10
2	90.08
3	91.94
4	92.98
5	93.60
6	94.42
7	95.45
8	97.11
9	97.52
10	95.80
Mean ± SD	93.70 ± 3.02
Min/Max	88.10/97.52

**Table 10:** Classification performance under multi-leaf perturbation scenarios.

Dataset/Scenario	Accuracy (%)	Precision (%)	Recall (%)	F1-Score (%)
Original single-leaf	97.00	97.14	97.06	97.04
Scenario 1 (same-direction neighbors)	95.20	96.11	95.04	95.16
Scenario 2 (opposite-direction neighbors)	96.07	96.18	96.05	95.95

**Table 11:** Performance comparison of the classification and regression frameworks (CNN and MLP) for MLC error identification.

Model	Learning Type	Spatial Feature Learning	Performance
CNN classifier	Classification	Yes	97.00%
MLP classifier	Classification	No	97.11%
CNN regression	Regression	Yes	MAE = 0.071 mm, Exact-match accuracy = 96.82%
MLP regression	Regression	No	MAE = 0.374 mm, Exact-match accuracy = 61.57%

## Data Availability

The datasets used and/or analysed during the current study are available from the corresponding author on reasonable request.

## Abbreviations

AI: Artificial intelligence; CNN: Convolutional neural network; DL: Deep learning; IMRT: Intensity-modulated radiation therapy; ML: Machine learning; MLC: Multi-leaf collimator; MU: Monitor unit; QA: Quality assurance; ROI: Region of interest; TPS: Treatment planning system; VMAT: Volumetric modulated arc therapy.

## References

[1] Otto K. (2008). Volumetric modulated arc therapy: IMRT in a single gantry arc. Med. Phys..

[2] Brahme A. (1988). Optimization of stationary and moving beam radiation therapy techniques. Radiother. Oncol..

[3] Ezzell G.A., Galvin J.M., Low D., Palta J.R., Rosen I., Sharpe M.B., Yu C.X. (2003). Guidance document on delivery, treatment planning, and clinical implementation of IMRT: Report of the IMRT Subcommittee of the AAPM Radiation Therapy Committee. Med. Phys..

[4] Fredh A., Scherman J.B., Fog L.S., Munck af Rosenschöld P. (2013). Patient QA systems for rotational radiation therapy: A comparative experimental study with intentional errors. Med. Phys..

[5] Heo T., Ye S.J., Carlson J., Park J.M. (2015). The effect of beam interruption during VMAT delivery on the delivered dose distribution. Phys. Medica.

[6] Park J.M., Park S.Y., Kim H. (2015). Modulation index for VMAT considering both mechanical and dose calculation uncertainties. Phys. Med. Biol..

[7] Park S.Y., Kim J.I., Oh D.H., Park J.M. (2019). Evaluation of the plan delivery accuracy of intensity-modulated radiation therapy by texture analysis using fluence maps. Phys. Medica.

[8] Heilemann G., Poppe B., Laub W. (2013). On the sensitivity of common gamma-index evaluation methods to MLC misalignments in Rapidarc quality assurance. Med. Phys..

[9] Kim J.I., Park S.Y., Kim H.J., Kim J.H., Ye S.J., Park J.M. (2014). The sensitivity of gamma-index method to the positioning errors of high-definition MLC in patient-specific VMAT QA for SBRT. Radiat. Oncol..

[10] Miften M., Olch A., Mihailidis D., Moran J., Pawlicki T., Molineu A., Low D.A. (2018). Tolerance limits and methodologies for IMRT measurement-based verification QA: Recommendations of AAPM Task Group No. 218. Med. Phys..

[11] Yan G., Liu C., Simon T.A., Peng L.C., Fox C., Li J.G. (2009). On the sensitivity of patient-specific IMRT QA to MLC positioning errors. J. Appl. Clin. Med. Phys..

[12] Park J.M., Park S.Y., Kim H., Kim J.H., Carlson J., Ye S.J. (2014). Modulation indices for volumetric modulated arc therapy. Phys. Med. Biol..

[13] Park S.Y., Park J.M., Kim J.I., Kim H., Kim I.H., Ye S.J. (2015). Textural feature calculated from segmental fluences as a modulation index for VMAT. Phys. Medica.

[14] Kim D.S., Kim S., Kang S.H., Kim T.H., Park S.H., Kim K.H., Suh T.S. (2018). To propose adding index of achievement (IOA) to IMRT QA process. Radiat. Oncol..

[15] Nyflot M.J., Thammasorn P., Wootton L.S., Ford E.C., Chaovalitwongse W.A. (2019). Deep learning for patient-specific quality assurance: Identifying errors in radiotherapy delivery by radiomic analysis of gamma images with convolutional neural networks. Med. Phys..

[16] Wootton L.S., Nyflot M.J., Chaovalitwongse W.A., Ford E. (2018). Error Detection in Intensity-Modulated Radiation Therapy Quality Assurance Using Radiomic Analysis of Gamma Distributions. Int. J. Radiat. Oncol. Biol. Phys..

[17] Kimura Y., Kadoya N., Tomori S., Oku Y., Jingu K. (2020). Error detection using a convolutional neural network with dose difference maps in patient-specific quality assurance for volumetric modulated arc therapy. Phys. Medica.

[18] Nakamura S., Sakai M., Ishizaka N., Mayumi K., Kinoshita T., Akamatsu S., Utsunomiya S. (2023). Deep learning-based detection and classification of multi-leaf collimator modeling errors in volumetric modulated radiation therapy. J. Appl. Clin. Med. Phys..

[19] Carlson J.N., Park J.M., Park S.Y., Park J.I., Choi Y., Ye S.J. (2016). A machine learning approach to the accurate prediction of multi-leaf collimator positional errors. Phys. Med. Biol..

[20] Osman A.F.I., Maalej N.M., Jayesh K. (2020). Prediction of the individual multileaf collimator positional deviations during dynamic IMRT delivery priori with artificial neural network. Med. Phys..

[21] Chuang K.C., Giles W., Adamson J. (2021). A tool for patient-specific prediction of delivery discrepancies in machine parameters using trajectory log files. Med. Phys..

[22] Klein E.E., Hanley J., Bayouth J., Yin F.F., Simon W., Dresser S., Holmes T. (2009). Task Group 142 report: Quality assurance of medical accelerators. Med. Phys..

